# Discussion Informed by Recurrent Lessons from a Systematic Review on Targeting Practices in Urban Humanitarian Crises

**DOI:** 10.1371/currents.dis.0d0be4b294b40f5e51ee7b58d9687ea0

**Published:** 2017-10-13

**Authors:** Ronak Patel, Jami King, Laura Phelps, David Sanderson

**Affiliations:** Department of Emergency Medicine, Stanford University, Stanford, California, United States; Duke University School of Law, Durham, North Carolina, United States; Norwegian Refugee Council, Oslo, Norway; Faculty of Built Environment, University of New South Wales, Sydney, New South Wales, Australia

## Abstract

**Introduction:**

Urbanization has challenged many humanitarian practices given the complexity of cities. Urban humanitarian crises have similarly made identifying vulnerable populations difficult. As humanitarians respond to cities with chronic deficiencies in basic needs stressed by a crisis, identifying and prioritizing the most in need populations with finite resources is critical.

**Methods:**

The full systematic review applied standard systematic review methodology that was described in detail, peer-reviewed, and published before the research was conducted.

Results: While the science of humanitarian practice is still developing, a systematic review of targeting vulnerable populations in urban humanitarian crises shed some light on the evidence base to guide policy and practice. This systematic review, referenced and available online, led to further findings that did not meet the pre-defined inclusion and exclusion criteria for evidence set out in the full review but that the authors, in their expert opinion, believe provide valuable insight nonetheless given their recurrence.

**Discussion:**

These additional findings that did not meet criteria for evidence and formal inclusion in the full manuscript, but deemed valuable by the subject expert authors, are discussed in this commentary

## Introduction

This discussion compliments a full systematic review by the authors published by Oxfam on targeting in urban humanitarian crises: What practices are used to identify and prioritize vulnerable populations affected by urban humanitarian emergencies? A systematic review. Oxford: Oxfam GB.^1^ Given the rapid growth of cities that outpace public services and extreme poverty marks some, sometimes large, portions of these cities. Even before a crisis, many urban dwellers may live below the Sphere minimum standards and needs can often outstrip the resources that humanitarian aid can bring to bear for disaster affected and non-affected persons in these cities. Identifying the most in need to prioritize for aid can be difficult. The goal of the systematic review was to evaluate the evidence base on targeting vulnerable populations in urban humanitarian crises. Urban vulnerability translates into multiple specific needs and a wide range of need assessment tools and methodologies were reviewed as long as they had been used in an urban humanitarian emergency and its performance had been evaluated. The evidence base was thin but some concrete findings could be drawn to guide practice.

In addition to the studies and findings reported in the full review, we identified 16 reports that were relevant to the topic of the review by meeting criteria for initial screening, but fell short of meeting the criteria for inclusion in the formal evidence-based synthesis. The predefined inclusion criteria for inclusion in the systematic review required that reports a) described a targeting practice had been employed in an urban humanitarian crisis and; b) that the practice was evaluated in some form or another. Standard systematic review methodology was used and a protocol for the systematic review was reviewed and published prior to the research. For the detailed methodology used in the systematic review including key terms of reference please refer to the original publication. As expert authors, we believe repeated lessons within these 16 reports provide valuable insights, reinforce the full systematic review findings or represent promising areas of research for further study despite their lack of supporting evidence as defined by the full systematic review methodology. This editorial commentary on targeting was focused around these recurrent lessons identified through the same thematic analysis used in the full systematic review. These summary commentary lessons from the full systematic review and these additional 16 reports about targeting approaches and other features of targeting are summarized in Table 1 and Table 2, respectively. These tables aim to provide a full accounting of our expert recommendations in an easily digestible format and represent a current 'state of the art' reflection of the our guidance on targeting practices for vulnerable populations in urban crises.

Given the overall lack of study, we argue that the absence of evidence does not necessarily make these lessons untrue. In fact, their repeated nature would actually qualify them as valid findings according to other criteria and they may eventually be supported by evidence. We included examples from the 16 reports within the commentary to provide a clearer depiction of the content discussed, not as anecdotal evidence.

**Commentary from Recurrent Lessons**

*Targeting ‘Affected’ vs. ‘Non-Affected’ populations does not work well*

In addition to targeting by displacement status, as highlighted in the full systematic review, targeting only persons affected by a crisis, those experiencing some loss as a result of the crisis, does not generally work well in the urban space even when resources are limited. Targeting only persons directly affected by the crisis such as those that suffer material or bodily harm directly by the crisis does not identify all or even the most vulnerable in urban humanitarian emergencies.^2^ To begin with, delineating who is ‘affected’ is difficult. Urban areas are complex systems in which a humanitarian emergency can have many indirect impacts on the ability to keep safe and obtain necessary goods and services making it difficult to truly define ‘affected or ‘non-affected.’ A flood may cause food prices to spike or foment violence that impacts households not directly damaged by floods themselves. Defining ‘affected’ is a critical challenge in this strategy. Additionally, chronically high levels of vulnerability within urban environments, sometimes themselves classified as (or otherwise meeting the criteria of) humanitarian crises, result in increased levels of baseline vulnerability among host or pre-crisis populations.^3^ Even after a crisis strikes, these poor but ‘non-affected’ populations (however defined), may be as or more vulnerable than persons that are directly or indirectly affected. The needs and vulnerability of ‘unaffected’ urban populations, especially the urban poor, may fall well within humanitarian imperatives and the goals of the targeting program. **Targeting by 'disaster affected persons does not work well in urban areas to accurately identify all or the most persons in need of a specific humanitarian aid interventions services.**

*Targeting upstream individuals and groups can enable recovery for the most vulnerable*

**Focusing on employers or individuals who are responsible for the livelihoods of others can be a means of returning beneficiaries to baseline livelihoods more quickly**.^4^ In their review of cash transfer programming across a variety of settings, IIED highlighted the importance of keeping in mind that the most-affected populations, and those vital to post-crisis recovery, may actually be the relatively better off, such as small business owners who employ lower income individuals.^5^

After the January 2010 earthquake in Haiti, targeting in one program focused on small business owners in an effort to help businesses remain in operation. Targeting focused on both the low-income as well as the middle-income groups “for two reasons: first, the middle groups were also badly affected by the earthquake and secondly, the services and activities carried out by the middle groups play a vital role in the recovery [of] the economy.”^6^ Focus groups in Haiti identified many vulnerable groups who were in need but who had not been targeted to receive services, including teachers. These focus groups identified the key link teachers provided in maintaining the human resources necessary for the education system and concluded that teachers should be prioritized as beneficiaries.^7^ **Incorporating this form of upstream targeting can facilitate smooth exit strategies and shifts the focus of targeting to engaging local actors.**

*Poverty is a useful but imperfect proxy for vulnerability when used alone*

Targeting vulnerability more generally based on poverty alone as a proxy may be appropriate in urban settings but requires detailed understanding to target correctly. **A snapshot of income or assets alone does not account for an accurate understanding of vulnerability.** As identified by MacAuslan and Farhat, poverty measures alone do not account for current or anticipated needs or the impact of a crisis on various socio-economic groups.^8^ Socio-economic variables including an assessment of coping strategies needs to be incorporated into targeting processes for more accurate targeting. Someone with less poverty may in fact be more vulnerable because they have more to lose or their means of living in the urban area are dependent on certain urban functions that can be impacted by a crisis or they may lack sufficient coping mechanisms. **Urban areas constitute an economic environment with goods and services driven by markets and thus poverty is key but does not wholly reflect vulnerability, which is multidimensional.**

*Protection concern may drive vulnerability more than other measures in some situations*

Despite having low vulnerability according to various indicators such as income or displacement status, concerns about safety may actually drive vulnerability in some cases. These protection concerns may override many other indicators of vulnerability.^9^ Protection concerns, however, may be linked to one of these specific indicators such as status as a refugee, residence within a conflict zone or engagement in child labour. For example, although a household may enjoy a higher socioeconomic status, female heads of household or specific ethnic groups may actually have limited access to basic goods and services due to constrained mobility from insecurity and fear of violence in the urban space. **Targeting approaches should add a protection lens to vulnerability analyses keeping in mind that safety and security can be paramount to wellbeing.**^10^

*Geographical targeting requires more detailed analysis given the density and heterogeneity of cities*

Geographic selection of urban areas to identify vulnerable populations by specific location, such as slums or low-lying coastal zones, can be a very effective process for targeting.^8^^,^^11^ However,** because many urban areas are not homogenous, care should be taken to identify geographic units that are smaller and not restricted to typical municipal or administrative boundaries.**^5^^,^^9^^,^^12^ Vulnerability is heterogeneous and ignorant of such boundaries in urban areas.

*Community participation and community based targeting are key to effective response but must be used with caution*

Many of the reports point toward the importance of community perspectives to help ensure that vulnerable areas and populations are not overlooked.^5^^,^^8^^,^^9^^,^^13^** Incorporating community perspectives can lead to locally derived measures of vulnerability** as described in the full systematic review. **Such locally derived measures, however, take time and resources to develop.** They may often lose comparability between contexts depending on their design. Yet comparable tools can be developed. For example, a scale based on local coping strategies can allow comparison if variations of coping can be applied to the same quantitative scale. These trade-offs must factor into their selection.

Community based targeting (CBT) or participatory targeting whereby the community directly identifies vulnerable persons or households is a growing practice in humanitarian interventions and compliments trends toward locally driven processes that are inclusive and suited for area based programming. The data from these practices is thin and of low quality but common lessons are repeated. In general,** incorporating community knowledge is clearly vital to good targeting, but participation can take many forms.**

**Lessons indicate the importance of having a nuanced understanding of the motivating factors driving individual participation in community-based targeting, as well as familiarity with local power dynamics and knowing whose voices are being heard.**^4^^,^^14^ As such, defining the community is critical and must be done carefully. Reports suggest that, in many ways, the community-based targeting practices relied upon in rural areas may not be relevant in urban contexts. Two reports highlighted the greater risk of using CBT in urban areas because close geographic proximity in urban environments may not necessarily indicate familiarity within communities as much as it may in rural areas, due to population mobility and fractured social networks.^5^^,^^15^ As a result, CBT that relies too heavily on community leaders or a small subset of people to identify beneficiaries can lead to biases and systematic exclusion of vulnerable groups or individuals more than in rural areas.^5^^,^^15^ Maintaining accuracy with community-based targeting requires triangulation and verification of information, as the most vulnerable neighbors may be unknown to or marginalized by community leaders.^16^ **Community engagement that unwittingly reinforces any pre-existing marginalization will do more harm than good.**

*Measuring food security an take many diverse forms but requires detailed analysis*

Papers on targeting for food insecurity were more numerous than other forms of sector-specific targeting, with 10 of the 21 evidence-based articles in the full systematic review dealing specifically with food security. Indeed, many of the evidence-based lessons and recurrent lessons above came from food security reports yet applied more generally as well. There was a lack of evidence directly comparing measures but a few insights are found in the various reports.

Targeting for food aid can measure a variety of characteristics such as consumption, purchasing power, access, nutritional status or coping strategies. These measures take multiple forms from a universal composite index to a locally derived context specific scale. All of these approaches come with their own advantages and disadvantages.^17^ Measuring nutritional intake or status provides only a snapshot of current or past consumption patterns. It does not accurately reflect food security, which is a latent property representing the ability to secure adequate food. Several other methods such as the Household Food Insecurity Assessment Scale attempt to do just that.^18^ By identifying a few key indicators, assessments may rapidly and efficiently identify vulnerable households and optimize targeting without extensive data. Although food prices play a large role in food consumption in urban areas, poverty alone is not always the best correlate for food insecurity. **To identify food insecure populations, understanding intra-household consumption patterns, capturing out of home consumption and the local coping strategies employed in additon to standard tools is key.**

*Involving local markets is important to targeting in urban areas while enhancing overall recovery*

Markets are important to addressing vulnerability in urban areas. Multiple reports point toward the importance of market analysis as urban food security is closely linked to commodity prices, income opportunities and wage rates.^19^ One key insight from Oxfam programming after the 2010 earthquake in Haiti was that “**a better understanding of crucial market chains can help lead to a more effective distribution of humanitarian resources, faster economic recovery and less risk of long term dependency on external assistance**.” The program in Haiti relied upon an inter-agency Emergency Market Mapping Analysis (EMMA) as well as a 2009 baseline assessment of income groups within Port Au Prince to create wealth group profiles for households affected by the earthquake and to inform targeting.^6^

**Programming aimed at improving market recovery post-crisis can also achieve the dual purpose of improving livelihoods as well as nutrition.** The review uncovered several studies that provide specific examples of how effective targeting involving local markets improved livelihoods. In Bulawayo, Zimbabwe, a program aimed at improving the nutritional status of vulnerable households successfully used local millers to produce, and existing retailers to sell, a low-cost maize alternative—sorghum.^20^ Oxfam used cash transfers in Nairobi to help households meet food needs through local markets and found that, along with other services to support entrepreneurship, the program enabled 50 per cent of beneficiary households to initiate, strengthen or restart a small business, creating more vibrant local markets.^21^ Additionally, an evaluation of the canteen program in Haiti after the 2010 earthquake found that 87% of beneficiaries who received small business grants were able to restart an economic activity and 64% of the women participants were able to restart their business because of the canteen program.^6^

Vulnerability can be invisible

**It is important to acknowledge that the most vulnerable persons may be those who try to stay invisible to authorities and even their neighbors**, and therefore may be missed by the targeting methods discussed in this review.^4^ This need for anonymity makes them particularly vulnerable. Also, a lack of representative leadership structures among refugee communities may result in individuals being overlooked or invisible to members of their own community.^22^ One proposed solution is to provide discreet community drop-in centres open to all that allow invisible persons to self-target for protection or service provision.^22^ Another proposed solution to connect with beneficiaries is to publicize hotlines where beneficiaries can call and receive information about services.^23^ All of the proposed solutions for this population require focused study.

**Figure:**
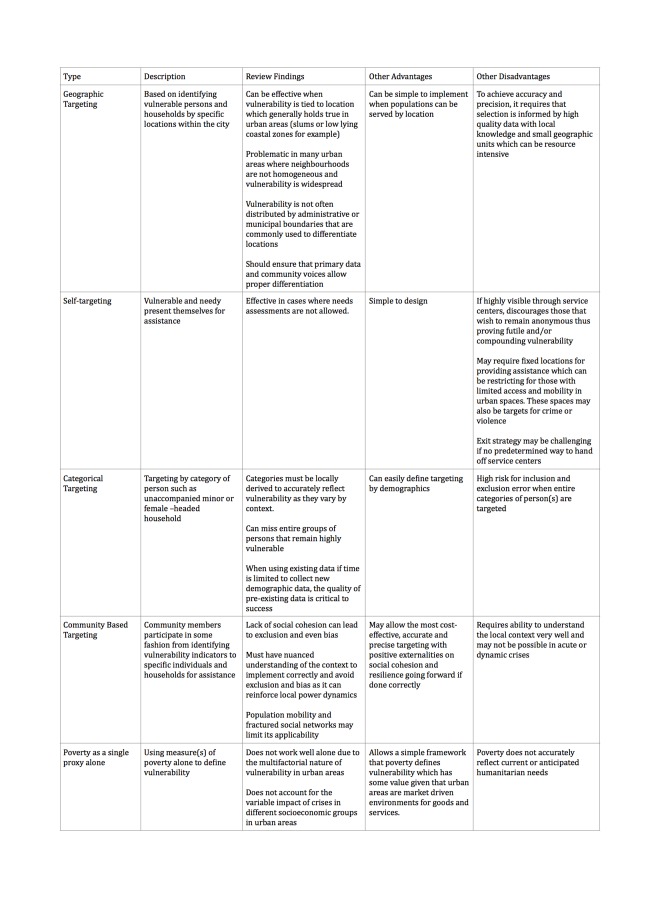
**Table 1:** Targeting strategies, review findings and other notable advantages and disadvantages to guide selection.

**Figure:**
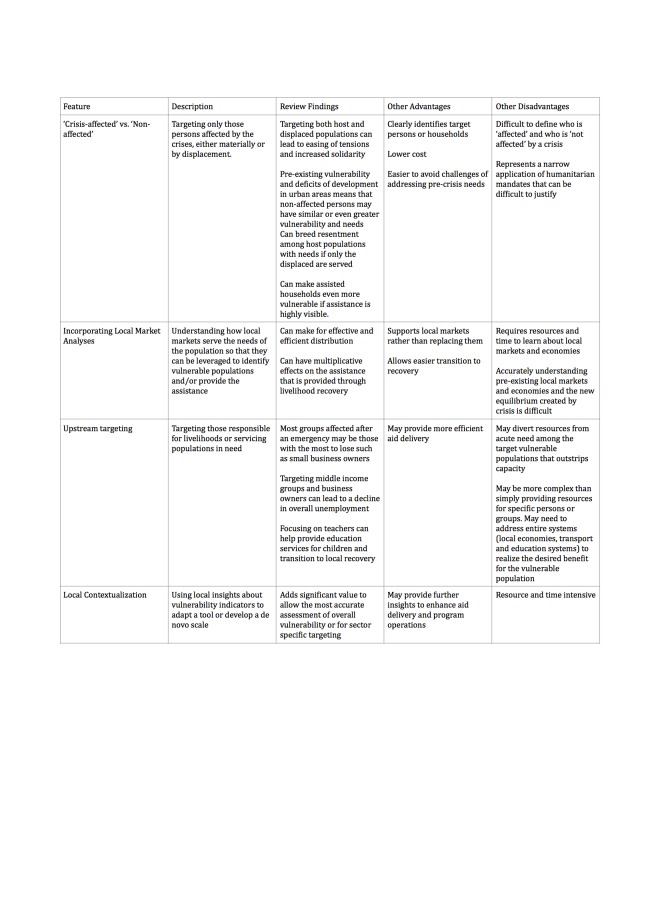
**Table 2:** Other features of targeting, review findings and notable advantages and disadvantages to guide selection.

## Conclusions and Recommendations

The current evidence and recurrent lessons above do not point to one single best approach for targeting vulnerable populations in urban crises. As humanitarian programs have a wide variety of goals and urban contexts and crises vary, the complex nature of vulnerability makes it impossible to have one best approach for each situation. Any approach will have strengths and limitations and none will be perfect as needs may often exceed available resources. In addition to these technical limitations, the security situation and political context can also impact the selection of a targeting approach. While evidence to guide humanitarian practice accumulates further debates on the humanitarian system and financing will have to progress in ways that incentivize evidence based practice. **Selection of targeting vulnerable populations in urban crises will have to weight the costs, benefits and feasibility of each approach and funding tied to evidence on how these three variables are assessed will be the best path moving forward.**

Vulnerability in urban areas is complex and interconnected such that assessing sector-specific vulnerability seems inappropriate or at least less useful. A person’s health and nutrition for instance is related to their shelter, access to sanitation, livelihood and surrounding security. **We believe the most vulnerable in urban humanitarian crises are best targeted using a collection of socioeconomic indicators along with in depth contextual understanding.**

Given that local actors, including government, will play a larger role in humanitarian response and the knowledge that municipal authorities and pre-existing organizations have, leveraging pre-existing data will be valuable. Often these may be incomplete, outdated or biased but improving these sources of information beforehand could be prove useful and efficient. As development and humanitarian practice come closer together within a resilience framework, reducing urban vulnerability as part of development efforts could help inform targeting in the event of a humanitarian crisis. **Practices for targeting vulnerable populations in urban crises should leverage urban development practice and tools in rapidly growing cities that aim to alleviate poverty and build resilience.**

Additionally, as the local expertise of already established actors and the affected community itself can prove invaluable, community based targeting (CBT) should be developed and tested further. The success of such an approach will depend on local capacity and technical expertise. Entrenched biases and power-dynamics may also bend a very well-intentioned approach into exacerbating vulnerability and a nuanced understanding of this context is required. The recent focus on area-based programming leans toward using a CBT approach as a key component. **Targeting approaches should exploit local knowledge and community based approaches with a nuanced understanding of these communities and power relations.**

Finally, the most promising approach may in fact be targeting based on methods that can be locally contextualized and rapidly so given the important of balancing accuracy with speedily delivering aid. As spaces within cities can be micro-environments that differ from neighbourhood to neighbourhood, locally contextualized tools should be further developed and expanded. **Research and practice should focus on developing and testing rapidly tools that can be quickly contextualized to the local situation.**

Overall, the full systematic review and this commentary from recurrent lessons lay bare the general lack of evidence guiding practice in targeting the most vulnerable in urban crises. Focused research and funding for it, as discussed in the full systematic review, must be prioritized to ensure humanitarian practice is grounded in rigorous evidence.

## Corresponding Author

Ronak B. Patel, rbpatel@gmail.com

## Data Availability

All relevant data are included within the paper.

## Competing Interests

The authors declare the following interest: DS serves on the Editorial Board for PLOS Currents: Disasters. He has not influenced or played any role in the peer-review, editorial decision making or publication of the manuscript.
